# The effect of antidepressant medication use on HbA1c levels in type 2 diabetes

**DOI:** 10.3389/fpsyt.2025.1693817

**Published:** 2026-01-12

**Authors:** Fatih Saglam, Necip Nas, Semih Saglik

**Affiliations:** 1 Department of Psychiatry, Siirt Training and Research Hospital, Siirt, Türkiye; 2 Department of Internal Medicine, Faculty of Medicine, Siirt University, Siirt, Türkiye; 3 Department of Radiology, Siirt Training and Research Hospital, Siirt, Türkiye

**Keywords:** antidepressants, glycemic control, HbA1c, psychiatric symptoms, type 2 diabetes

## Abstract

**Background:**

Chronic psychological symptoms such as anxiety and depression are known to negatively affect glycemic control in patients with type 2 diabetes (T2D). However, the impact of psychiatric treatment on glycemic outcomes remains underexplored.

**Objective:**

This study aimed to evaluate the effect of psychiatric treatment on HbA1c levels in T2D patients presenting with psychological symptoms.

**Methods:**

A total of 64 T2D patients with psychological complaints were retrospectively analyzed and divided into two groups based on psychiatric treatment status: 39 patients received antidepressant therapy, while 25 did not. Demographic data, comorbidities, and HbA1c levels before and after treatment or during follow-up were compared between groups. Statistical analyses were performed to assess differences in HbA1c changes.

**Results:**

There were no significant differences between groups regarding age, gender, or comorbid medical conditions (p>0.05). Patients receiving psychiatric treatment showed a significant reduction in HbA1c levels compared to those untreated (37.5% vs. 9.4% HbA1c decrease, p<0.001). Conversely, HbA1c levels increased in a higher proportion of untreated patients (28.1% vs. 15.6%). The reduction in HbA1c was more pronounced in individuals with baseline HbA1c levels above 8%. Patients treated with SSRIs, particularly sertraline and escitalopram, showed notable reductions in HbA1c levels.

**Conclusion:**

Our study suggests that antidepressant therapy in patients with depressive symptoms may contribute to reductions in HbA1c levels. This finding supports the notion that targeted treatment of depression can positively influence glycemic control. These results underscore the importance of integrating depression screening and management into T2D care strategies. Further longitudinal and large-scale studies are warranted to confirm these findings and clarify the mechanisms underlying the psychometabolic effects of antidepressants.

## Introduction

Type 2 diabetes (T2D) is a disease characterized by hyperglycemia resulting from a relative or absolute impairment in insulin secretion, accompanied by varying degrees of resistance to the action of insulin (1). It is one of the most common chronic diseases worldwide, with risk factors including genetics, obesity, hypercholesterolemia, poor dietary habits, sedentary lifestyle, smoking, and high birth weight (2). Hemoglobin A1c (HbA1c) is the most frequently used parameter in the monitoring of T2D. Sustained elevations in HbA1c increase the risk of diabetes-related complications, which in turn may further hinder or delay patients’ adherence to treatment (1, 2).

Prolonged stress and anxiety can contribute to insulin resistance and increased cardiovascular risk over time (3, 4). Persistent psychiatric symptoms in depressive and anxiety disorders may impair glycemic control in patients with T2D (5). Depression comorbid with DM is linked to higher mortality within three years, indicating it as an independent risk factor (6). Although less studied, anxiety in DM is also associated with poor metabolic outcomes and increased complications but lacks a defined treatment approach (7). Common anxiety disorders include Generalized Anxiety Disorder, Panic Disorder, and Social Phobia. Additionally, personality traits such as anxiety and negativity can negatively affect treatment adherence (8).

Antidepressant treatments are primarily classified into several pharmacological groups: Selective Serotonin Reuptake Inhibitors (SSRIs: sertraline, escitalopram, citalopram, fluoxetine, paroxetine, fluvoxamine), Serotonin-Norepinephrine Reuptake Inhibitors (SNRIs: venlafaxine, duloxetine), Tricyclic Antidepressants (TCAs: clomipramine, imipramine, amitriptyline), and Monoamine Oxidase Inhibitors (MAOIs: selegiline) (9). TCAs and MAOIs are generally not preferred as first-line agents due to their higher likelihood of adverse effects. Improvement in psychiatric symptoms begins within 4 to 8 weeks following the initiation of antidepressant therapy (9).

The presence of chronic stress and psychiatric symptoms is now widely recognized as being associated with elevated HbA1c levels, which in turn contributes to an increased risk of diabetes-related complications and mortality (10–14). However, there is currently no standardized preventive strategy or treatment approach targeting this issue. The aim of this study is to investigate the effect of psychiatric treatment on glycemic control, as measured by HbA1c levels, in patients diagnosed with T2D who present with psychiatric symptoms, thereby evaluating the potential benefits of addressing mental health issues in the management of T2D.

## Materials and method

### Ethical approval

The study protocol was reviewed and approved by the Ethics Committee of Siirt University (Approval No. 137230, Date: April 21, 2025). In accordance with the Declaration of Helsinki, all participants provided informed consent before their inclusion in the study. The lead investigator thoroughly explained the study objectives and treatment procedures to each participant, ensuring that they fully understood the use of their data for research purposes. This process confirmed that all participants voluntarily agreed to participate and to the use of their data in the study.

### Study populations

A total of 64 patients diagnosed with T2D, who were being followed in the Internal Medicine/Endocrinology outpatient clinic and presented with psychiatric complaints (such as anxiety, panic attacks, depression, or adjustment disorders) were included in the study. Between January 1 and December 1, 2024, 40 patients received psychiatric outpatient care. The required sample size was calculated to ensure adequate statistical power. Based on an assumed effect size of 0.74 (Cohen’s d), a 95% confidence level, and a 5% margin of error, at least 37 patients in the treatment group and 25 patients in the control group were needed. Consequently, 39 patients were included in the study; one patient that did not come for follow-up was not included in the study. The patients’ comorbidities included hypertension; cardiovascular diseases such as coronary artery disease and arrhythmias; neurological disorders including cerebrovascular accidents (stroke), headache syndromes, and neuropathy; as well as liver conditions like hepatic steatosis and hyperlipidemia. To reflect real-world clinical practice, the study included patients receiving antidepressants regardless of concomitant psychiatric medications, as polypharmacy is common. In addition, psychiatric symptoms other than depression can also lead to increased stress hormones and blood sugar irregularities. Recruitment was not limited solely to patients with a formal depression diagnosis because antidepressants are frequently prescribed for subclinical depressive symptoms, anxiety disorders and other psychiatric disorders in T2D, and including these patients allows for a more representative assessment of antidepressant effects on glycemic control. Both the antidepressant-treated and untreated diabetes patient groups received comparable antidiabetic therapy. During the follow-up period, neither the type nor the dosage of antidiabetic medications was changed.

### Measures

#### Diagnosis of T2D

The diagnosis of T2D in the study participants was established based on the diagnostic criteria outlined by the American Diabetes Association (ADA) (15). According to these guidelines, a diagnosis of T2D was confirmed if at least one of the following criteria was met (1): fasting plasma glucose ≥126 mg/dL after at least 8 hours of fasting; (2) 2-hour plasma glucose ≥200 mg/dL during a 75 g oral glucose tolerance test (OGTT); (3) a random plasma glucose ≥ 200 mg/dL in the presence of classic symptoms of hyperglycemia or hyperglycemic crisis; or (4) a glycated hemoglobin (HbA1c) level ≥ 6.5%. All diagnoses were confirmed retrospectively using patients’ clinical records and laboratory data. Furthermore, to ensure diagnostic stability, only individuals with a confirmed diagnosis of T2D for at least six months and who were under regular follow-up by internal medicine or endocrinology clinics were included in the study. Psychiatric symptoms were required to be present for at least one month with mild-to-moderate functional impairment, and patients had not received psychiatric treatment within the previous year. All individuals with T2D were outpatients under routine endocrinology follow-up, with a diabetes duration of approximately 2–5 years.

#### Diagnostic evaluation of mental health disorders

A total of 39 patients who had been examined by a psychiatrist and diagnosed according to the Diagnostic and Statistical Manual of Mental Disorders, Fifth Edition (DSM-5) criteria published by the American Psychiatric Association were included in the study (16). The psychiatric diagnoses included Generalized Anxiety Disorder (GAD), Panic Disorder, Major Depressive Disorder (MDD), adjustment disorder, and somatoform disorder. Generalized Anxiety Disorder is characterized by excessive and uncontrollable worry occurring almost daily for at least six months. Panic Disorder is defined by recurrent, unexpected panic attacks accompanied by anticipatory anxiety and fear lasting for at least one month. Somatoform Disorder involves persistent physical symptoms accompanied by excessive thoughts, feelings, and behaviors related to those symptoms. Major Depressive Disorder is marked by persistent sadness and anhedonia, often accompanied by low energy, pessimism, impaired concentration, and disturbances in sleep and appetite. Adjustment Disorder is characterized by emotional or behavioral symptoms emerging in response to a stressful or traumatic life event. Obsessive-Compulsive Disorder (OCD), although less commonly diagnosed in this cohort, is defined by intrusive thoughts and repetitive behaviors aimed at reducing associated distress. Antidepressant medications, primarily SSRIs and SNRIs, are commonly used as first-line agents in both the initiation and maintenance phases of treatment for these conditions.

### The inclusion criteria

Although psychiatric disorders are diverse, this study focused on antidepressant use because depression is the most common mental health condition in patients with T2D and is known to influence glycemic control. i) Age ≥18 years; ii) Diagnosis of T2D; iii) Presence of psychiatric symptoms (e.g., anxiety, depression, panic attacks, adjustment disorder) identified during routine clinical evaluation; iv) Availability of at least two HbA1c measurements during the follow-up period; v) For the treatment group: receipt of antidepressant therapy at a therapeutic dose for at least 4 weeks.

### The exclusion criteria

i) Use of antipsychotic or mood stabilizer medications; ii) Initiation or change in antidiabetic medications during the follow-up period; iii) Presence of acute medical illness or hospitalization within the study period; iv) Incomplete medical records or missing HbA1c data v) Participants diagnosed with intellectual developmental disorder.

### Data collection and follow-up

HbA1c values were retrospectively reviewed for all patients. HbA1c levels were measured before and after the initiation of antidepressant therapy. A minimum observation period of 1.5 months was maintained for all 39 treated patients to consistently assess the potential impact of antidepressant use on glycemic control. For the untreated group, two HbA1c values were selected: one at the time psychiatric symptoms were documented and another obtained during follow-up. An effort was made to ensure an average interval of three months between the two HbA1c measurements; however, a minimum interval of 1.5 months was required to allow sufficient time for the antidepressant treatment to take effect. During the follow-up period, no changes were made to the patients’ antidiabetic regimens in terms of medication type or dosage (15).

Although HbA1c typically reflects glycemic status over an approximately three-month period, intervals shorter than three months (minimum 1.5 months) were included due to variability in real-world clinical follow-up. Antidepressants generally begin to exert clinical effects within 4–6 weeks, which may allow early metabolic changes to emerge during this period. Nonetheless, only 4 of the 39 patients had follow-up HbA1c measurements within 1.5–2 months, while the remainder were assessed at three months or longer. Therefore, the inclusion of a small subset with shorter intervals is acknowledged as a methodological limitation.

HbA1c determination was based on HPLC (Variant Turbo II, Bio-Rad Laboratories, Inc., CA, USA).

### Statistical analysis

The data analyses of this study were performed using SPSS version 20.0 (Statistical Package for the Social Sciences, Chicago, IL). Categorical variables were expressed as frequencies (n) and percentages (%), while continuous variables were presented as mean ± standard deviation (SD). For statistical evaluation, the Student’s t-test was used to compare normally distributed variables between groups, whereas the Mann-Whitney U test was applied for variables that did not follow normal distribution. Depending on sample size, the Chi-square test or Fisher’s exact test was used for comparisons of categorical variables. A p-value of less than 0.05 was considered statistically significant.

## Results

A total of 64 patients were included in the study, of whom 52 (81.3%) were female and 12 (18.8%) were male. Patients were categorized into two groups based on psychiatric treatment status: Group 1 (n=39) received psychiatric treatment, and Group 2 (n=25) did not receive psychiatric treatment (**Table 1**). There were no statistically significant differences between the groups in terms of age, gender distribution, or presence of comorbid medical conditions (p > 0.05) (**Table 2**).

**Table 1:** Sociodemographic characteristics and clinical profiles of patients.

Patient no	Age	Gender	Psychiatric disorder	Additional medical disorder
1	57	Female	Adjustment Disorder	Hypertension
2	56	Female	Panic Disorder	
3	77	Female	Adjustment Disorder	Rhythm Disorder
4	45	Male	Adjustment Disorder	
5	65	Female	Generalized Anxiety Disorder	Coronary Artery Disease
6	55	Female	Adjustment Disorder	
7	72	Female	Generalized Anxiety Disorder	Hypertension
8	77	Female	Generalized Anxiety Disorder	Hypertension
9	57	Male	Adjustment Disorder	Hypertension
10	63	Male	Adjustment Disorder	Cerebrovascular Disease
11	53	Male	Panic Disorder	Hypertension
12	31	Female	Generalized Anxiety Disorder	Ulcerative Colitis
13	52	Male	Adjustment Disorder	COPD
14	63	Female	Panic Disorder	Asthma
15	65	Female	Generalized Anxiety Disorder	Hepatitis B
16	51	Female	Adjustment Disorder	Kidney Disease
17	44	Female	Depression	
18	53	Female	Generalized Anxiety Disorder	Rhythm Disorder
19	56	Female	Somatoform Disorder	Hypertension
20	53	Female	Adjustment Disorder	Hepatitis B
21	60	Male	Generalized Anxiety Disorder	Cerebrovascular Disease
22	61	Female	Generalized Anxiety Disorder	
23	48	Female	Generalized Anxiety Disorder	Hypertension
24	77	Female	Depression	Hypertension
25	61	Female	Generalized Anxiety Disorder	
26	64	Female	Generalized Anxiety Disorder	
27	60	Female	Adjustment Disorder	
28	25	Female	Depression	Migraine
29	70	Female	Adjustment Disorder	Hypertension
30	73	Female	Generalized Anxiety Disorder	
31	62	Female	Generalized Anxiety Disorder	Coronary Artery Disease
32	59	Male	Generalized Anxiety Disorder	
33	57	Male	Depression	Hypertension
34	51	Female	Generalized Anxiety Disorder	Hypertension
35	61	Female	Adjustment Disorder	
36	19	Female	Depression	
37	26	Female	Adjustment Disorder	
38	60	Female	Generalized Anxiety Disorder	Fibromyalgia
39	55	Female	Generalized Anxiety Disorder	Coronary Artery Disease
40	32	Male	Generalized Anxiety Disorder	
41	55	Female	Generalized Anxiety Disorder	Hypertension
42	68	Female	Depression	Hypertension
43	23	Male	Adjustment Disorder	
44	61	Female	Generalized Anxiety Disorder	Coronary Artery Disease
45	65	Female	Panic Disorder	
46	70	Female	Generalized Anxiety Disorder	Coronary Artery Disease
47	67	Female	Generalized Anxiety Disorder	Hypertension
48	54	Female	Generalized Anxiety Disorder	
49	26	Male	Depression	
50	56	Female	Somatoform Disorder	Hypertension
51	69	Female	Depression	Hypertension
52	59	Female	Panic Disorder	Hypertension
53	50	Female	Depression	
54	58	Female	Adjustment Disorder	Fibromyalgia
55	53	Female	Somatoform Disorder	
56	45	Female	Somatoform Disorder	
57	53	Female	Generalized Anxiety Disorder	Myasthenia Gravis
58	68	Male	Obsessive Compulsive Disorder	Prostatic Hyperplasia
59	77	Female	Generalized Anxiety Disorder	Hypertension
60	58	Female	Panic Disorder	Hypertension
61	84	Female	Somatoform Disorder	Hypertension
62	67	Female	Adjustment Disorder	Coronary Artery Disease
63	25	Female	Adjustment Disorder	
64	47	Female	Adjustment Disorder	

Patients 1–39 received antidepressant treatment; patients 40–64 did not receive antidepressants.

**Table 2:** Comparison of clinical and demographic characteristics between groups.

	Group 1 (Psychiatric treatment received)	Group 2 (Psychiatric treatment not received)	Total	P
Number of patients, n	39	25	64	
Age ± SD (Years)	56.25 ± 13.41	55.60 ± 15.84	56.00 ± 14.29	0.859
Gender, n (%) - Female - Male	31 (48.4%) 8 (12.5%)	21 (32.8%) 4 (6.3%)	52 (81.3%) 12 (18.8%)	0.751
Change in HbA1c, n (%) - Decreased - Increased - Unchanged	24 (37.5%) 10 (15.6%) 5 (7.8%)	6 (9.4%) 18 (28.1%) 1 (1.6%)	30 (46.9%) 28 (43.8%) 6 (9.4%)	**0.001**
Comorbid Medical Condition, n (%) - Present - Absent	26 (40.6%) 13 (20.3%)	15 (23.4%) 10 (15.6%)	41 (64.1%) 23 (35.9%)	0.390

Bold values indicate statistically significant results.

Comparison of HbA1c changes between groups revealed a significant difference (p < 0.001). In Group 1, 37.5% of patients demonstrated a decrease in HbA1c levels, while only 9.4% of patients in Group 2 showed a decrease. Conversely, 28.1% of untreated patients experienced an increase in HbA1c compared to 15.6% in the treated group (**Table 2**).

Among patients receiving psychiatric treatment, the medications administered included sertraline (n=16), escitalopram oxalate (n=12), fluoxetine (n=3), paroxetine (n=3), duloxetine (n=3), citalopram (n=1), and venlafaxine (n=1). Detailed HbA1c measurements before and after treatment, alongside outcomes by treatment status, are summarized in **Table 3**. Additionally, **Table 4** presents comprehensive HbA1c data and outcomes specifically for the untreated group. Sixteen patients were treated with sertraline, and a reduction in HbA1c levels was observed in 12 of them. Twelve patients were treated with escitalopram oxalate, with HbA1c reduction observed in 5 patients.

**Table 3:** Comprehensive HbA1c measurements and outcomes by group according to treatment status.

Patient no	Initial HbA1c	Psychiatric medication used	Final HbA1c	HbA1c change	Outcome
1	6,0	Sertraline	5,5	-0,5	Decreased
2	7,0	Escitalopram	7,0	0,0	Unchanged
3	7,5	Sertraline	7,7	0,2	Increased
4	6,2	Sertraline	5,4	-0,8	Decreased
5	9,5	Sertraline	8,4	-1,1	Decreased
6	8,0	Sertraline	6,3	-1,7	Decreased
7	6,7	Sertraline	6,0	-0,7	Decreased
8	6,0	Duloxetine	6,3	0,3	Increased
9	6,7	Sertraline	6,2	-0,5	Decreased
10	5,2	Sertraline	5,1	-0,1	Decreased
11	6,1	Escitalopram	5,9	-0,2	Decreased
12	6,6	Escitalopram	6,0	-0,6	Decreased
13	8,5	Sertraline	9,9	1,4	Increased
14	8,6	Escitalopram	8,4	-0,2	Decreased
15	6,6	Duloxetine	6,4	-0,2	Decreased
16	8,1	Sertraline	7,7	-0,4	Decreased
17	6,4	Escitalopram	6,8	0,4	Increased
18	6,1	Escitalopram	8,1	2,0	Increased
19	7,5	Duloxetine	7,5	0,0	Unchanged
20	7,2	Fluoxetine	7,7	0,5	Increased
21	7,9	Escitalopram	6,4	-1,5	Decreased
22	7,4	Escitalopram	7,1	-0,3	Decreased
23	6,5	Sertraline	6,5	0,0	Unchanged
24	5,8	Escitalopram	6,1	0,3	Increased
25	6,4	Escitalopram	5,6	-0,8	Decreased
26	7,4	Paroxetine	7,0	-0,4	Decreased
27	8,5	Fluoxetine	6,4	-2,1	Decreased
28	14,6	Venlafaxine	11,1	-3,5	Decreased
29	7,4	Sertraline	5,5	-1,9	Decreased
30	6,2	Sertraline	6,0	-0,2	Decreased
31	8,9	Sertraline	10,8	1,9	Increased
32	6,4	Escitalopram	6,4	0,0	Unchanged
33	11,5	Sertraline	8,3	-3,2	Decreased
34	8,0	Citalopram	11,7	3,7	Increased
35	8,2	Escitalopram	10,2	2,0	Increased
36	9,0	Paroxetine	8,4	-0,6	Decreased
37	8,9	Sertraline	8,8	-0,1	Decreased
38	8,8	Paroxetine	7,7	-1,1	Decreased
39	6,6	Fluoxetine	6,6	0,0	Unchanged

**Table 4:** Comprehensive HbA1c measurements and outcomes in the untreated group.

Patient no	Initial HbA1c	Final HbA1c	HbA1c change	Outcome
40	10,4	12,9	2,5	Increased
41	7,7	9,1	1,4	Increased
42	8,6	10,0	1,4	Increased
43	9,3	10,6	1,3	Increased
44	6,8	6,4	-0,4	Decreased
45	11,4	11,1	-0,3	Decreased
46	9,4	10,5	1,1	Increased
47	10,8	10,7	-0,1	Decreased
48	9,0	10,5	1,5	Increased
49	7,0	7,0	0,0	Unchanged
50	6,0	6,7	0,7	Increased
51	6,5	7,1	0,6	Increased
52	5,8	5,9	0,1	Increased
53	12,0	12,7	0,7	Increased
54	9,1	11,0	1,9	Increased
55	7,0	8,0	1,0	Increased
56	7,8	7,2	-0,6	Decreased
57	9,6	9,9	0,3	Increased
58	10,8	10,6	-0,2	Decreased
59	10,8	10,9	0,1	Increased
60	7,8	8,1	0,3	Increased
61	9,3	10,2	0,9	Increased
62	6,4	6,8	0,4	Increased
63	9,7	8,8	-0,9	Decreased
64	10,0	10,9	0,9	Increased

## Discussion

HbA1c is a widely used marker for monitoring glycemic control and predicting diabetes-related complications and mortality (6). The most important finding of this study is that psychiatric treatment in patients with T2D significantly improves glycemic control. Patients who received pharmacological treatment primarily with SSRIs such as sertraline, escitalopram, fluoxetine, paroxetine, and other antidepressants like duloxetine and venlafaxine showed a significant reduction in HbA1c levels. In contrast, patients without psychiatric treatment exhibited stable or increased HbA1c levels. This glycemic improvement occurred independently of age, sex, or comorbid medical conditions. These findings underscore the potential metabolic benefits of psychiatric treatment and emphasize the importance of integrating mental health care into the management of T2D. Although many studies have highlighted the benefits of addressing mental health in diabetes management, evidence remains limited regarding the impact of antidepressant use on glycemic control in patients with T2D. This study aims to address this gap. However, given the potential risks associated with antidepressant use in individuals with T2D, a clearer understanding of prescribing practices in this population is needed, particularly in primary health care.

Our study demonstrated that psychiatric treatment with antidepressants, particularly SSRIs and SNRIs, significantly improved glycemic control in patients with T2D, as evidenced by a notable reduction in HbA1c levels compared to untreated patients. It should be noted that patients in the treatment group had lower baseline HbA1c and higher rates of well-controlled glycemia (<7%). Further reductions in HbA1c in these patients may provide limited additional benefits and could potentially increase the risk of hypoglycemia. Conversely, small increases within the well-controlled range (e.g., from 6.0% to 6.3%) are unlikely to be clinically meaningful. These considerations highlight the importance of interpreting HbA1c changes in the context of baseline glycemic control when evaluating the effects of antidepressant therapy. These results are supported by existing literature: a meta-analysis of randomized controlled trials found that SSRIs improved glycemia, with a pooled effect size of –0.34 for HbA1c compared to placebo (95% CI –0.48 to –0.21; p < 0.001), and specifically fluoxetine and escitalopram showed significant reductions in HbA1c (–0.29 and –0.33 respectively). SSRIs seem to have an association with improvement in glycemia, which is not moderated by depression status, diabetes status, or change in weight across studies (17). Another umbrella review indicated that citalopram and escitalopram appear to exert beneficial effects on glycemic control, as evidenced by reductions in HbA1c and FBG (18). Furthermore, a Canadian primary care cohort study of over 1,000 diabetic patients demonstrated numerically lower post-treatment HbA1c ratios among users of escitalopram, venlafaxine, and other selected antidepressants compared to citalopram. This study can inform future research examining the relationship between antidepressants and blood glucose and provides insight into the limitations pertaining to the use of health data in health research (19). Proposed mechanisms underlying these metabolic benefits may include improvement in depressive symptoms leading to better medication adherence and self-care behaviors, as well as possible direct pharmacological effects on glucose metabolism, such as enhanced insulin sensitivity and reduced hypothalamic-pituitary-adrenal axis hyperactivity seen with SSRIs (20). Khapre et al. (20) presented a moderate level of evidence that antidepressants treatment among depressed diabetic population leads to improved glycemic control. There is no significant difference in pooled FBG, weight, and BMI measured at the end of study in antidepressant and placebo group. Their results support the emphasis on early recognition and prompt pharmacological treatment and control of depression in T2D population to achieve better glycemic control.

In the present study, 16 patients were treated with sertraline, and a reduction in HbA1c levels was observed in 12 of them. In a recent study, 72 out of 225 patients diagnosed with T2D were also diagnosed with depression, and HbA1c levels in these individuals were shown to be associated with depressive symptoms (10). In that study, 70 patients received sertraline at doses of 50–100 mg/day, and after six months, a reduction in depressive symptoms was reported. Conversely, a 2018 clinical study involving 33 patients with coexisting T2D and depression administered sertraline over a three-month period. Changes in body weight, body mass index (BMI), HbA1c, and fasting/postprandial glucose levels were assessed. Unlike our findings, no significant changes in HbA1c levels were observed; however, there was a significant reduction in both body weight and BMI (11). Antidepressants included in the study (e.g., duloxetine, venlafaxine, sertraline) may exert different metabolic effects; however, due to the limited size, drug or class specific subgroup analyses could not be performed. As a result, all antidepressants were evaluated collectively, which may mask potential differences between agents. Future studies with larger cohorts are needed to assess class- and drug-specific metabolic outcomes.

In the present study, 12 patients were treated with escitalopram oxalate, and HbA1c reduction was observed in 5 of them. A previous study investigating the glycemic effects of escitalopram reported significant reductions in fasting glucose, postprandial glucose, and HbA1c levels after 12 weeks of treatment in 40 patients, suggesting beneficial effects on both mood and glycemic control in individuals with T2D (12). Three patients received paroxetine and three received fluoxetine; HbA1c levels decreased in all three patients on paroxetine and in one patient on fluoxetine. However, in a study with 56 diabetic patients, while paroxetine effectively reduced depression and anxiety symptoms, it had no significant effect on HbA1c levels (21). Another study comparing paroxetine and fluoxetine similarly found no significant impact on HbA1c despite improvements in psychiatric symptoms (22).

Recent *in vitro* evidence has also shown that both paroxetine and sertraline may directly stimulate insulin secretion, promote β-cell proliferation, and reduce apoptosis, potentially contributing to improved glycemic outcomes (23). A meta-analysis of 24 studies on fluoxetine concluded that it may aid in glycemic control among individuals with glucose metabolism disorders and assist with weight management in obese patients (24). One subject enrolled in the present study was treated with citalopram. Although data on citalopram are more limited compared to other SSRIs, it has been reported to effectively reduce depressive symptoms in diabetic patients without negatively affecting glycemic control (25). Four patients received SNRIs (three duloxetine, one venlafaxine), and HbA1c reduction was observed in two of them. Duloxetine is commonly used in diabetic patients, particularly for peripheral neuropathy and pain, either as an alternative to or in combination with gabapentin and pregabalin (26). A recent 2025 study reported that while pregabalin and gabapentin were more effective for neuropathic pain, duloxetine demonstrated superior glycemic control. The mean HbA1c levels in that study were 9.42 for gabapentin, 10.43 for pregabalin, and 7.72 for duloxetine (27). Similarly, venlafaxine has been shown to be a viable option for diabetic neuropathy due to its minimal side effect profile (28). However, despite its effectiveness in reducing depressive and anxiety symptoms, venlafaxine has been associated with increased HbA1c levels (from 7.64 to 8.03) and potential weight gain (29, 30). Our findings align with the network meta-analysis by Srisurapanont et al. (31), which showed that while SSRIs and SNRIs are effective in reducing depressive symptoms in patients with T2D, their impact on glycemic control varies by drug type. Limited evidence from short-term trials in depressed patients with T2D suggests that escitalopram and agomelatine may have a favorable profile in reducing depression and controlling glycemic goals (31). In our study, SSRIs like sertraline were associated with improved HbA1c levels, supporting their potential benefit in both mood and metabolic regulation. A recent within-subject before-after study demonstrated that the initiation of antidepressant therapy, particularly with SSRIs, was associated with a modest reduction in HbA1c levels in patients with T2D (32). These results support the potential dual benefit of antidepressants on both glycemic control and mental health in diabetic populations. These findings underscore the importance of individualized antidepressant selection in patients with T2D, considering both psychiatric efficacy and metabolic effects. Further longitudinal studies are warranted to clarify the long-term impact of specific agents on glycemic control.

### Limitations of the study

This study has several limitations. The small sample size and retrospective design restrict generalizability and precludes causal inference. The relatively short follow-up period may not fully capture long-term glycemic or psychiatric outcomes. Heterogeneity in antidepressant type and dosage further limits comparability. Key clinical variables such as fasting glucose, body weight, medication adherence, dietary and lifestyle factors, psychiatric severity measures, and glycemic variability were not assessed, reducing the comprehensiveness of the analysis. Additionally, the lack of propensity score matching, confounder adjustment, or multivariate regression prevents adequate control of baseline differences, including BMI, diabetes duration, insulin use, and comorbidities. Another limitation is the inability to determine the proportion of patients achieving the guideline-recommended HbA1c target (<7%) due to incomplete documentation of glycemic goal attainment. Future studies with larger samples, standardized treatment protocols, and more rigorous statistical and clinical assessments are needed to validate these findings.

## Conclusions

HbA1c trajectory remains a key parameter in the global management of T2D, serving as a strong predictor of complications. Our findings specifically focus on the effects of antidepressant therapy on glycemic outcomes. Treatment with SSRIs, such as escitalopram, paroxetine, and fluoxetine, was associated with improved HbA1c in certain patients, although responses varied by agent. SNRIs, particularly duloxetine, showed potential benefits in both neuropathic pain management and HbA1c reduction. Given the heterogeneity of responses and the multifactorial nature of T2D, personalized selection of antidepressants based on metabolic profile, psychiatric symptoms, and comorbid conditions may optimize both psychiatric and diabetic outcomes. These results underscore the importance of integrated care models, especially in primary care settings where mental health and metabolic parameters are monitored concurrently. Further large-scale, prospective studies are warranted to confirm these findings and elucidate the mechanisms by which antidepressants influence glycemic control in patients with comorbid T2D and depression.

## Data Availability

The raw data supporting the conclusions of this article will be made available by the authors, without undue reservation.
